# Combination of Liposomal CpG Oligodeoxynucleotide 2006 and Miltefosine Induces Strong Cell-Mediated Immunity during Experimental Visceral Leishmaniasis

**DOI:** 10.1371/journal.pone.0094596

**Published:** 2014-04-14

**Authors:** Rahul Shivahare, Preeti Vishwakarma, Naveen Parmar, Pawan Kumar Yadav, Wahajul Haq, Mrigank Srivastava, Suman Gupta, Susanta Kar

**Affiliations:** 1 Division of Parasitology, CSIR-Central Drug Research Institute, Lucknow, India; 2 Division of Medicinal and Process Chemistry, CSIR-Central Drug Research Institute, Lucknow, India; National Center for Cell Science, India

## Abstract

Immuno-modulators in combination with antileishmanial drug miltefosine is a better therapeutic approach for treatment of Visceral Leishmaniasis (VL) as it not only reduces the dose of miltefosine but also shortens the treatment regimen. However, immunological mechanisms behind the perceived benefits of this combination therapy have not been investigated in detail. In the present study, we hypothesized that potential use of drugs that target the host in addition to the parasite might represent an alternative strategy for combination therapy. We investigated immune responses generated in *Leishmania donovani* infected animals (hamsters and mice) treated with combination of CpG-ODN-2006 and miltefosine at short dose regimen. Infected animals were administered CpG-ODN-2006 (0.4 mg/kg, single dose), as free and liposomal form, either alone or in combination with miltefosine for 5 consecutive days and parasite clearance was evaluated at day 4 and 7 post treatment. Animals that received liposomal CpG-ODN-2006 (lipo-CpG-ODN-2006) and sub-curative miltefosine (5 mg/kg) showed the best inhibition of parasite multiplication (∼97%) which was associated with a biased Th1 immune response in. Moreover, compared to all the other treated groups, we observed increased mRNA expression levels of pro-inflammatory cytokines (IFN-γ, TNF-α and IL-12) and significantly suppressed levels of Th2 cytokines (IL-10 and TGF-β) on day 4 post treatment in animals that underwent combination therapy with lipo-CpG-ODN-2006 and sub-curative miltefosine. Additionally, same therapy also induced heightened iNOS mRNA levels and NO generation, increased IgG2 antibody level and strong T-cell response in these hamsters compared with all the other treated groups. Collectively, our results suggest that combination of lipo-CpG-ODN-2006 and sub-curative miltefosine generates protective T-cell response in an animal model of visceral leishmaniasis which is characterized by strong Th1 biased immune response thereby underlining our hypothesis that combination therapy, at short dose regimen can be used as a novel way of treating visceral leishmaniasis.

## Introduction

Visceral leishmaniasis (VL) is a vector-borne fatal infection usually caused by protozoan parasites *Leishmania donovani* and *L. infantum*. VL threatens 200 million people in 62 countries with an estimated 5, 00,000 new cases and 60,000 deaths each year [1]. The risk of mortality in VL has recently increased due to it's association with HIV infection [2]. It is one of the most neglected parasitic diseases in terms of drug development and there are no licensed vaccines available in the market. At present, VL treatment relies on a handful number of drugs such as pentavalent antimonials, amphotericin-B and its formulations, paromomycin and the only orally administered drug miltefosine [3]. However, none of these drugs are ideal for treatment due to their high toxicity, resistance issues, prohibitive prices, long treatment regimen and mode of administration [4]. Over the past few decades significant improvements have been made in the number of treatments available for VL, with both new drugs and new formulations of old drugs that have been either recently approved or are in clinical trials are now available [5], [6]. Recently, combination therapy using immunomodulators with standard antileishmanial compounds have become increasingly popular and several studies have reported benefits of co-administration of antileishmanial drugs with immunostimulants as they shorten the course of treatment, delay or prevent the emergence of resistance and increase the efficacy of current therapeutic regimen [7], [8], [9], [10]. Imiquimod, a novel immune response activating compound approved by USA Food and Drug Administration (FDA) is currently being used with paromomycin for successful treatment of cutaneous leishmaniasis (CL) [11]. Quassin [12], fucoidan [13], and curdlan [14] are other examples of immunomodulators that have recently been explored for their potential to kill the *Leishmania* parasites by boosting host immunity in experimental models of VL. Since, progression of VL infection is generally associated with down regulation of the host immune system, *Leishmania* has evolved several skills to inactivate macrophage immune functions to survive inside the cells (Olivier et al, 2005, 15). The outcome of infection depends on the production and/or secretion of immunosuppressive molecules that includes, transforming growth factor (TGF)-β, interleukin (IL)-10 and prostaglandin E2 (PGE2) [15], [16]. These molecules distort the normal immune response by suppressing host-protective microbicidal molecules, including cytokines like interferon (IFN)-γ, IL-1, IL-12, and tumor necrosis factor-α (TNF-α), and reactive nitrogen and oxygen species (RNS and ROS) [15]–[17]. Growing body of evidences suggest that compounds/agents that boost host cell activation by Th1 biased immune response might be useful as potential therapeutic agents for treatment of experimental VL [11]–[14]. Synthetic oligodeoxynucleotides (ODN) containing unmethylated cytosine phosphate guanine (CpG) motifs mimic microbial DNA and are recognized by toll-like receptor 9 on B-cells, dendritic cells (DCs), natural killer (NK) cells and monocytes [18], [19]. They are known to stimulate innate immune responses that induce macrophage to secrete IL-12, which in turn induces IFN-γ secretion by NK cells [20]. Use of CpG-ODNs as vaccine adjuvant have also been extensively studied and it has been observed that during *in vivo* conditions free CpG-ODNs are rapidly eliminated from circulation due to adsorption and degradation, however their encapsulation in liposomes provides improved protection from rapid degradation and also enhances their protective efficacy [21], [22].

We have previously explored the therapeutic efficacy of various combinations of CpG-ODN-2006 and miltefosine at sub-curative doses for the treatment of experimental VL [23]. In the present study, we explored immunological basis of protective immune responses observed in Syrian golden hamsters and mice that were treated with different formulations of CpG-ODN-2006 alone or in combination with miltefosine in a short term treatment regimen.

## Material and Methods

### Parasite

The WHO reference strain of *L. donovani* (MHOM/IN/80/Dd8), originally obtained from Imperial College, London (UK) was maintained in the laboratory as promastigotes *in vitro* in M-199 medium (Sigma-Aldrich, USA) supplemented with 10% FBS at 24°C±2°C and *in vivo* in golden hamsters (*Mesocricetus auratus*) from amastigote to amastigote by serial passage [23].

### Ethics statement

The study was carried out in strict accordance with the recommendations of Committee for the Purpose of Control and Supervision of Experiments on Animals (CPCSEA), New Delhi, India. Usage of animals were approved by the Institute's Animal Ethical Committee (IAEC) and all the experiments were conducted in compliance with the Institutional Animal Ethics Committee (IAEC) of CSIR-CDRI for use and handling of animals (IAEC approval no: 27/2005/PARA/IAEC).

### Animals

Inbred hamsters (40–45 g) and Balb/c mice (20–22 g) of both sexes were used for the study. Animals were bred in the National Animal Laboratory Centre, housed in CSIR-CDRI, Lucknow. For experimental studies, animals were housed in climate-controlled (23±2°C; relative humidity: 60%) and photoperiod-controlled (12 h light-dark cycles) animal quarters. They were fed standard rodent pellets supplemented with grain and had free access to drinking water.

### CpG-ODN-2006 and Miltefosine

A fully phosphothioated class B oligodeoxynucleotide, CpG-ODN-2006 (MW 7,698) and ODN was purchased from Trilink Biotechnologies Inc., USA. Miltefosine was purchased from SynphaBase AG, Switzerland. For *in vivo* treatment, CpG-ODN-2006 and miltefosine were dissolved in phosphate buffer saline (PBS).

### Liposome preparation

Liposomes consisting of dipalmitoylphosphatidylcholine (DPPC) and cholesterol were prepared by the dehydration-rehydration vesicle (DRV) method as described previously [23]. The lipid phase consisting of DPPC (6 µM), and cholesterol (2 µM) was dissolved in chloroform: methanol (2∶1, v/v) in a flat bottom tube and the solvent was removed by slow evaporation under nitrogen with deposition of a thin film of lipid on the tube surface. The tube was dried to remove any traces of solvent and the thin dry lipid film was dispersed in a solution of appropriate quantity of CpG-2006 in PBS and vortexed and the suspension was sonicated for 1 min in an ultrasonicator. The concentration of encapsulated CpG-2006 into liposomes was determined using UV absorption spectrophotometer at 260 nm.

### Infection and treatment of animals

Golden hamsters and Balb/c mice were infected with 1×10^7^ amastigotes suspended in PBS via intracardiac route or lateral tail vein respectively [23]. Since the infection is well adapted in these animals and establishes itself within 15 days, nine groups of hamsters and Balb/c mice, each consisting of five animals was used for experiments. Pre-treatment splenic biopsy of all hamsters and autopsy of two Balb/c mice was carried out to assess the degree of infection. Following biopsy impression smears made on slides, fixed in methanol, stained with 10% Giemsa stain in PBS and the number of amastigotes/1000 cell nuclei of liver or spleen was counted. Animals with +1 infection (5–10 amastigotes/100 spleen or liver cell nuclei) were included in the studies. Drug treatment was initiated two days after assessing the degree of infection either via intraperitoneal (IP) or oral route (PO). Animals in group I received PBS and served as normal controls. Animals in group II were infected and left untreated while animals in group III received free-CpG-ODN-2006 (0.4 mg/kg, single dose) by IP route, in group IV they received liposomal CpG-2006 (0.4 mg/kg, single dose, IP), in group V they received free-CpG-ODN-2006 (0.4 mg/kg, single dose, IP) + sub-curative miltefosine (5 mg/kg×5 days, oral), in group VI they received Lipo-CpG-ODN-2006 (0.4 mg/kg, single dose, IP) + sub-curative miltefosine (5 mg/kg×5 days, oral), in group VII received sub-curative dose of miltefosine (5 mg/kg×5 days, oral) and in group VIII they received curative dose of miltefosine (40 mg/kg for hamsters and 20 mg/kg for Balb/c mice × 5 days) by oral route and in group IX received control ODN (0.4 mg/kg, single dose, IP). To assess the therapeutic efficacy, animals were sacrificed at day 4 and 7 post-treatment and the number of amastigotes/1000 cell nuclei was counted in giemsa stained impression of liver and spleen tissue from different experimental group. Total parasite load in each organ is expressed in LDU as described by Stauber et al [24]. 1 LDU  =  amastigote per nucleated cell × organ weight in milligram.

### Real time RT-PCR analysis for estimation of cytokines and iNOS levels

For quantification of cytokines and iNOS levels, total RNA was isolated from splenocytes of both mice and hamsters using RNeasy kit (Qiagen, USA) as described previously [25]. RNA (1 µg) was used as a template for cDNA synthesis using the SuperScript first strand synthesis system (Invitrogen, USA) and primer sequences used for quantification of hamster cytokines (TNF-α, IFN-γ, IL-12, IL-10 and TGF-β) and iNOS are given in Table 1 [26]. Primer sequences for mice were designed as described previously [27]. PCR reactions were performed in triplicates using ABI Power SYBR Green PCR Master mix on Roche Applied Science light cycler 480.0 instrument using software version 1.5.0. Relative quantification of each target gene were normalized to the housekeeping gene HGPRT or GAPDH mRNA level and expressed as a fold change compared with uninfected control using the comparative cycle threshold (CT) method.

**Table 1:** Sequences of forward and reverse primers used for quantitative real time-PCR.

Number	Primer	Primer sequence
1	HGPRT forward	5′ GATAGATCCACTCCCATAACTG 3′
	HGPRT reverse	5′ TACCTTCAACAATCAAGACATTC 3′
2	IFN-γ forward	5′ GCTTAGATGTCGTGAATGG 3′
	IFN-γ reverse	5′ GCTGCTGTTGAAGAAGTTAG 3′
3	IL-12 forward	5′ TATGTTGTAGAGGTGGACTG 3′
	IL-12 reverse	5′ TTGTGGCAGGTGTATTGG 3′
4	TNF-α forward	5′ TTCTCCTTCCTGCTTGTG 3′
	TNF-α reverse	5′ CTGAGTGTGAGTGTCTGG 3′
5	iNOS forward	5′ CGACGGCACCATCAGAGG 3′
	iNOS reverse	5′ AGGATCAGAGGCAGCACATC 3′
6	IL-10 forward	5′ TGCCAAACCTTATCAGAAATG 3′
	IL-10 reverse	5′ AGTTATCCTTCACCTGTTCC 3′
7	TGF-β forward	5′ ACGGAGAAGAACTGCTGTG 3′
	TGF-β reverse	5′ GGTTGTGTTGGTTGTAGAGG 3′

### Preparation of soluble *L. donovani* promastigote antigen (SLA)

Soluble *L. donovani* promastigote antigen (SLA) was prepared as described previously [28]. Briefly, late log phase promastigotes (10^9^) were harvested from 3 to 4 days of culture, washed 4 times in cold PBS and resuspended in PBS containing protease inhibitors cocktail (Sigma, USA). Resuspended sample was subjected to rapid freeze-thawing (−70°C and 37°C) for 6 times in liquid nitrogen followed by 5 min incubation on ice. Partially lysed promastigotes were then subjected to ultrasonication for 3 periods with pulse of 30 s each (Soniprep 150; MSE, UK) in ice. The suspension was centrifuged at 10,000 rpm for 30 min at 4°C. The protein content of the supernatant containing soluble antigen was estimated by Bradford assay and stored at −70°C.

### Measurement of nitric oxide (NO) production

Splenocytes from different experimental groups of Balb/c mice and hamsters were incubated with or without SLA for SLA (5 µg/mL for mice and 50 µg/mL for hamsters) for 3 days in 5% CO_2_ incubator at 37°C [29]. NO generation was quantified by measuring accumulation of nitrite in culture supernatants by Griess reagent, nitrite concentrations were calculated by comparing with a standard curve generated by sequentially diluting sodium nitrite in deionized water.

### Lymphoproliferative responses from normal, *L. donovani* infected and treated hamsters

Lymphocyte proliferation was assessed by using the XTT lymphocyte proliferation kit (Biological industries, Israel) instead of [^3^H] thymidine [26]. Mesentric lymph node cells of hamsters were isolated on day 4 and day 7 post treatment and allowed to proliferate (1×10^5^ cells) for 3 days in the presence and absence of mitogen, concanavalin A (ConA, 5 µg/mL) and for 5 days in case of soluble *Leishmania* antigen (SLA, 10 µg/mL) [29]. Eighteen hours prior to termination of experiment, XTT reagent was added to each well and absorbance was measured with a spectrophotometer (Bio-Tek instruments, USA) at 480 nm with 650 nm being the reference wavelength.

### Determination of *Leishmania* specific IgG and its isotypes IgG1 & IgG2 antibody response by ELISA

Sera from mice and hamsters belonging to different experimental groups were collected on day 4 and day 7 post treatment and assayed for antileishmanial IgG and its isotypes IgG1 and IgG2 antibody response as described earlier [30], [31]. Briefly, microtiter plates were coated overnight with SLA (2 µg/mL for hamsters and 0.5 µg/mL for mice) at 4°C. Nonspecific binding sites were blocked by adding 2% bovine serum albumin in PBS, and the plates were washed with PBS containing 0.05% Tween 20. After washing, hamster's sera (1∶100) was added and incubated for 2 h at RT. After washing, plates were incubated with biotin-conjugated mouse anti-Armenian and Syrian hamster IgG, IgG1 and biotinylated anti-Syrian hamster IgG2 antibodies (1∶1000) (BD Biosciences, USA) for 1 h at RT. Thereafter, plates were further incubated with peroxidase-conjugated streptavidin (1∶1000) (BD Biosciences, USA) for another 1 h. For mice, sera were added at 1∶1000 dilution, followed by washing of cells and incubation with peroxidase-conjugated goat anti-mouse IgG1 or IgG2a antibodies (BD Pharmingen, San Diego, CA) for 1 h [31]. Finally, substrate o-phenylenediamine dihydrochloride (Sigma-Aldrich, USA) was added and absorbance was measured at 492 nm using an ELISA reader.

### T-cell proliferation assay

T-cell proliferation assay was performed as described earlier [32]. Briefly, splenocytes from different experimental groups of Balb/c mice and hamsters was prepared after Ficoll density gradient centrifugation and then suspended in complete RPMI 1640. Cells were plated in triplicate at 10^5^ cells/well concentration in 96-well plates and allowed to proliferate for 3 days at 37°C in 5% CO2 incubator in either in the presence or absence of SLA (5 µg/mL for both mice and hamsters). ConA (Sigma Aldrich, St. Louis, MO) was used as a positive control and the mitogen was added at a concentration of 5 µg/mL. At 18 h before harvesting, cells were pulsed with 1 µCi of [^3^H] thymidine/well. Incorporation of [^3^H] thymidine, as an index of proliferation, was measured using a liquid scintillation counter (Tri-Carb 2100TR; Packard Instrument.

### Statistical analysis

Data shown are representative of three sets of independent experiments having five animals per group. Each experiment was performed in triplicate and the results were analyzed by one way ANOVA followed by Tukey's post test using graph pad Prism (version 5.0). Differences in *P* values of <0.05 between treatment groups were considered significant.

## Results

### Antileishmanial efficacy of sub-curative miltefosine is enhanced by lipo-CpG-2006 through host-protective cytokine response and NO generation

**Figure 1:**
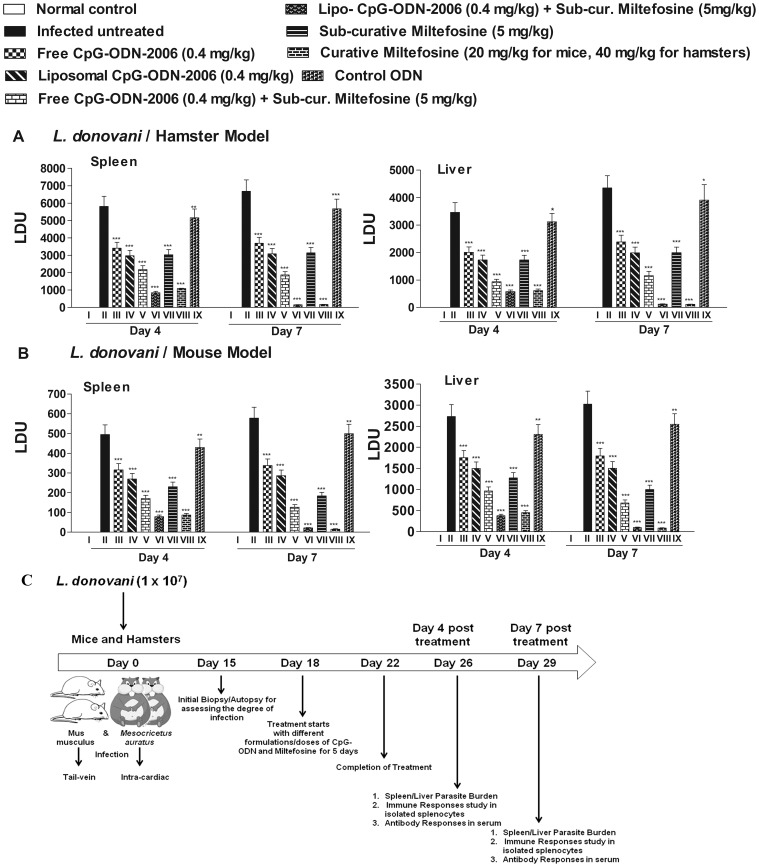
Effect of different formulation/combination of CpG-ODN-2006 and miltefosine on parasite burden. (A) *L. donovani*-infected mice and (B) hamsters were treated with various combinations of CpG-ODN-2006 and miltefosine as described in materials and methods section. At day 4 and day 7 post treatment (A) hamsters and (B) mice of different experimental groups were sacrificed and the hepatic as well as the splenic parasite load was determined by stamps-smear method. Total parasite load in each organ is expressed in LDU unit. 1 LDU  =  amastigote per nucleated cell × organ weight in milligram. (C) Schematic representation of the experimental protocol for treatment of *Leishmania donovani* infected mice/hamsters with different formulation/combination of CpG-ODN-2006 and miltefosine. Data represents here are representative of three independent experiments. Each of the experiments was done a minimum of three times and data represents mean ± SD. The significance between different experimental groups was calculated by one way ANOVA followed by Tukey's post test using graph pad Prism (version 5.0). Significance: Group II vs normal and all treated groups, group V vs VI and group V vs VII (*P<0.05, **P<0.01, ***P<0.001 and ns; non-significant).

We previously demonstrated that administration of single dose of lipo-CpG-ODN-2006 (1 nM or 0.4 mg/kg) along with sub-curative dose of miltefosine is a novel therapeutic approach for treatment of VL at short dose regimen [23]. Experimental infection with *L. donovani* in Balb/c mice represent acute form of VL whereas the same in hamsters represent chronic form of human VL, so we confirmed inhibition of organ parasite load in both rodent model of each treated group on day 4 and day 7 post treatment. Combination of lipo-CpG-2006 and sub-curative miltefosine showed the best and almost similar inhibition of parasite burden in *L. donovani* infected Balb/c mice and hamsters on day 7 post treatment (Figure 1A and 1B). The animals treated with lipo-CpG-ODN-2006 plus sub-curative miltefosine (Group VI) showed significantly reduced level of splenic and hepatic parasite load (Group VI >86% and >84% reduction in liver and splenic parasite load for mice and >83% and >85% reduction in liver and splenic parasite load for hamsters in comparison to infected untreated control) at day 4 post treatment which was similar to high dose curative miltefosine treated group (Group VIII, >83% and >82% reduction in liver and splenic parasite load for mice and >82% and >81% reduction in liver and splenic parasite load for hamsters in comparison to infected untreated control i.e. group II) (Figure 1A and 1B). However, reduction of parasite burden was best as observed at day 7 post treatment, where group VI showed >96% reduction in both liver and splenic parasite burden for hamsters and >97% reduction in both liver and splenic parasite burden for mice. This was even comparable with animals treated with curative dose miltefosine (Group VIII) which showed >97% reduction in both liver and splenic parasite burden in both rodent model. Administration of free liposomes in infected Balb/c mice and hamsters did not have any effect on parasite inhibition (data not shown). Whereas administration of control ODN (without CpG motifs, Group XI) showed little reduction in organ parasite burden (10.3% and 11.8% reduction in liver and splenic parasite burden for hamsters and 13.6% and 15.8% reduction in both liver and splenic parasite burden for mice at day 7 post treatment in comparison to infected control). Moderate suppression of organ parasite burden was found at in infected animals treated with free or lipo CpG-ODN-2006, sub-curative miltefosine and free CpG-ODN-2006 plus sub-curative miltefosine (≈35–77% reduction in organ parasite burden for both rodent model in Group III, IV, V and VII in comparison to group II) at day 7 post treatment. These results prompted us to investigate the detailed cellular immunological responses following combination therapy in both chronic (*L. donovani*/hamster model) and acute (*L.donovani*/Balb/c mouse model) form of VL to better understand the antileishmanial effector mechanism. We used the same groups of hamsters and mice as mentioned above (i.e. day 4 and day 7 post treated, infected and untreated controls) for various immunological studies except Group XI which showed little suppression of parasite burden as compared to other treated group.

### Combination therapy induces host protective cytokine response and NO generation

**Figure 2:**
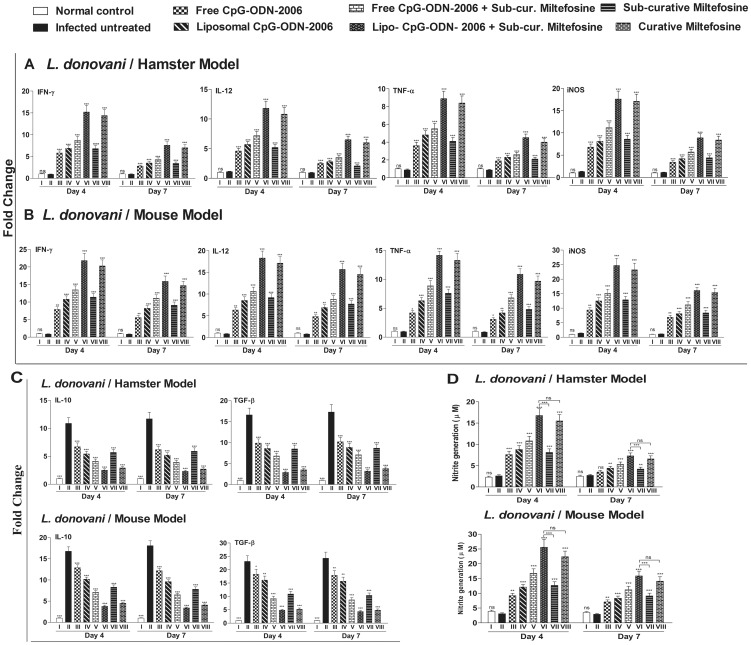
Effect of CpG-ODN-2006 and miltefosine combination therapy on cytokine response, iNOS expression and NO generation. Animals (mice and hamsters) were infected with *L. donovani* and treated with various combinations of CpG-ODN-2006 and miltefosine as described in figure legend 1. (A and B) RNA were isolated from isolated splenocytes on day 4 and day 7 post treatments and levels of mRNA expression of Th1 cytokines (IFN-γ, IL-12 and TNF-α) and iNOS (A) as well as Th2 cytokines (IL-10 and TGF-β) (B) were evaluated by real-time PCR. The mRNA level were normalized to HGPRT and GADPH for hamsters and Balb/c mice respectively and expressed as a fold change compared with uninfected control animals. (C) Nitrite generation by supernatant of splenocytes derived from different experimental groups of animals were stimulated with SLA (5 µg/mL for both hamsters and mice) for 72 h and assayed as described in materials and methods section. Data represents here are representative of three independent experiments. Each of the experiments was done a minimum of three times and data represents mean ± SD. The significance between different experimental groups was calculated by one way ANOVA followed by Tukey's post test using graph pad Prism (version 5.0). Significance: Group II vs normal and all treated groups, group VI vs VII and group VI vs VIII (*P<0.05, **P<0.01, ***P<0.001 and ns; non-significant).

Development of strong cell-mediated immunological responses, like Th1 biased cytokine responses, NO generation and down regulation of Th2 cytokines is associated with disease resolution in VL. Since CpG-ODNs are powerful inducers of cellular immune response [20], we firstly explored the underlying immune response in hamster model of chronic VL that were treated with free and lipo-CpG-ODN-2006 either alone or in combination with miltefosine. We investigated mRNA transcript levels of Th1/Th2 cytokines in different treated group and found that at day 4 post treatment IFN-γ, IL-12 and TNF-α were significantly increased in all treated group as compared to infected untreated hamsters (Group II) with maximum elevation (16.7, 10.7 and 10.1 fold, respectively) in lipo-CpG-ODN-2006 plus sub-curative miltefosine treated group (Group VI) (Figure 2A). This was even greater as compared to the hamsters treated with curative dose of miltefosine (Group VIII, 15.8, 9.8 and 9.5 fold higher than infected control, respectively) (Group VIII). The elevation continued even at day 7 post treatment albeit at diminished level (Figure 2A). Similar trend was also observed in Balb/c mouse model, where infected mice treated with lipo-CpG-ODN-2006 plus sub-curative miltefosine showed pronounced Th1 dominance (Group VI, expression of IFN-γ, IL-12 and TNF-α mRNA transcripts were 24.5, 22.6 and 15.5 fold higher respectively than infected untreated ones) at day 4 post treatment (Figure 2B). This Th1 biased immune response was also reflected in downstream induction of iNOS, the enzyme responsible for generation of NO, a potent macrophage-derived microbicidal molecule. Similar to Th1 cytokines, maximum induction of iNOS mRNA transcript and NO generation was observed in lipo-CpG-ODN-2006 plus sub-curative miltefosine treated group of mice and hamsters (Group VI) at both day 4 and 7 post treatment (13.5 and 8.1 fold higher expression of iNOS than infected untreated hamsters, respectively and 17.6 and 14.6 fold higher than infected untreated Balb/c mice, respectively) (Figure 2A and 2B). Treatment of *Leishmania* infected hamsters with lipo-CpG-ODN-2006 plus sub-curative miltefosine (Group VI) led to a maximum (p<0.001) generation of nitric oxide (16.8±1.6 and 7.3±0.7 µM) at day 4 and day 7 post treatment, respectively which was >6 fold and 2.6 fold higher than infected untreated hamsters (Group II, 2.6 and 2.7 µM respectively) (Figure 2D). The NO generation by splenocytes of this group of hamsters (Group VI) was significantly (p<0.001) and much higher than the group of hamsters which were treated with sub-curative dose of miltefosine alone (Group VII, 8.1±0.8 and 4.2±0.4 µM at day 4 and day 7 post treatment, respectively) as well as even higher than animals treated with curative dose of miltefosine (Group VIII, 15.5±1.6 and 6.6±0.7 µM at day 4 and day 7 post treatment respectively) (Figure 2D). Similarly in Balb/c mice, significantly higher generation of NO was observed in group VI when compared with group VII and even superior than curative miltefosine treated group (Group VIII) (Figure 2D). Contrary to these observations, *Leishmania* infected hamsters and Balb/c mice that were left untreated (Group II) showed a remarkable increase in mRNA transcripts of IL-10 and TGF-β which were significantly (p<0.001) decreased in all treated groups at same time points (Figure 2C). Here too, we observed maximum reduction in Group VI at both day 4 and 7 post treatment. Taken collectively, increased expression of Th1 cytokines and iNOS and lower expression of Th2 cytokines in group VI animals highlight that shorter dose regimen of sub-curative miltefosine along with single shot of 0.4 mg/kg of lipo-CpG-ODN-2006 has the potential to induce better host protective cellular immune responses than curative dose of miltefosine.

### Combination therapy significantly increases host T-cell proliferation

**Figure 3:**
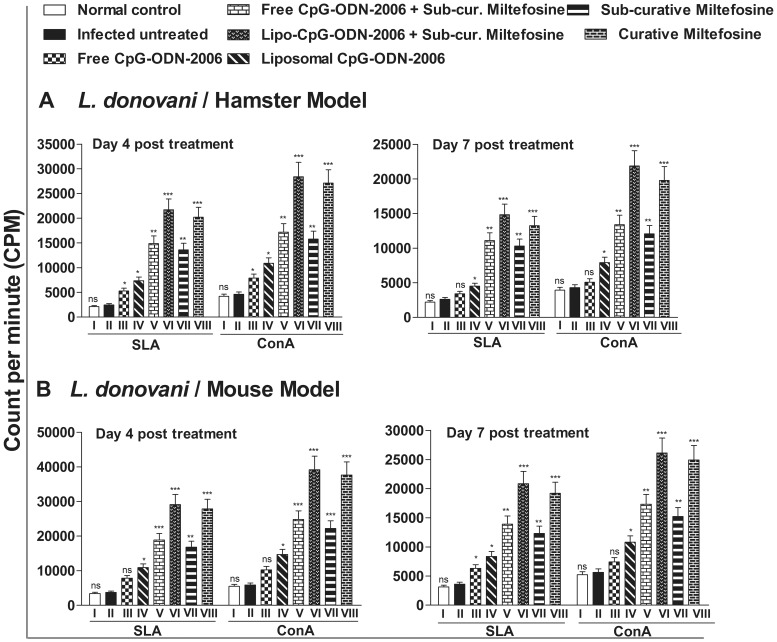
Effect of CpG-ODN-2006 and miltefosine combination therapy on T-cell proliferation at day 4 and 7 post treatments. Splenocytes were isolated from all the groups of (A) hamsters (normal, infected and treated) and (B) mice followed by incubation in the presence and absence of concanavalin A (5 µg/mL) or soluble *Leishmania* antigen (5 µg/mL for both hamster and for mice) at 37°C for 72 h and T-cell proliferation was assessed by incorporation of ^3^H-thymidine. Three independent experiments were done and each bar represents pooled data (mean ± SD) of five animals and the data represent the means of triplicate wells. Mean ± SD between different groups were calculated by one way ANOVA followed by Tukey's post test using graph pad Prism (version 5.0). Significance: Group II vs normal and all treated groups (*P<0.05, **P<0.01, ***P<0.001 and ns; non-significant).

*Leishmania* specific lymphocyte proliferation greatly reduces the parasite burden and promotes healing in infected patients [33]. However, cellular immune machinery and T-lymphocyte proliferation is critically impaired during VL [16], [30] which could be reversed by successful therapy. After observing the potential therapeutic and immunostimulatory effect in terms of reduction in organ parasite burden and enhancement of Th1 biased immune responses, we became interested to investigate whether the T-cell anergy observed during progressive infection could be reversed by the combination therapy. We therefore performed T-cell proliferation assay where the cellular proliferative responses of isolated splenocytes of all groups of animals were measured against the mitogen (i.e., ConA) and SLA. As shown in Figure 3A (Group II), infected hamsters treated with combination of lipo-CpG-ODN-2006 and miltefosine (Group VI) showed maximum T-cell-proliferative responses on day 4 and 7 post treatment, respectively, when stimulated with ConA as well as SLA. The comparative analysis with untreated group (Group II) showed (6.1 and 5.09) fold higher T-cell proliferation (p<0.001) when stimulated with ConA and (8.7 and 5.6) fold higher T-cell proliferation (p<0.001) when stimulated with SLA at day 4 and day 7 post treatment, respectively (Figure 3A). This was higher than hamsters treated with curative dose of miltefosine (Group VIII). Moderately significant T-cell proliferations were recorded in hamsters treated with free-CpG-ODN-2006 (Group III, p<0.05), lipo-CpG-ODN-2006 (Group IV, p<0.05), free-CpG-ODN-2006 plus miltefosine (Group V, p<0.01) and sub-curative miltefosine alone (Group VII, p<0.01) groups at day 4 post treatment in both ConA and SLA stimulated cells (Figure 3A). Interestingly, the status of antileishmanial T-cell repertoire in infected and all treated group were essentially similar regardless of rodent model as we observed parallel outcome of splenocyte proliferation in all treated group of Balb/c mice where Group VII showed maximum T-cell proliferation (6.6 and 4.5 fold higher T-cell proliferation (p<0.001) when stimulated with ConA and 7.8 and 5.7 fold higher T-cell proliferation (p<0.001) when stimulated with SLA in comparison to infected untreated Balb/c mice at day 4 and day 7 post treatment, respectively) (Figure 3B).

### Combination therapy modulates *Leishmania*- specific IgG and its isotypes in infected hamsters

**Figure 4:**
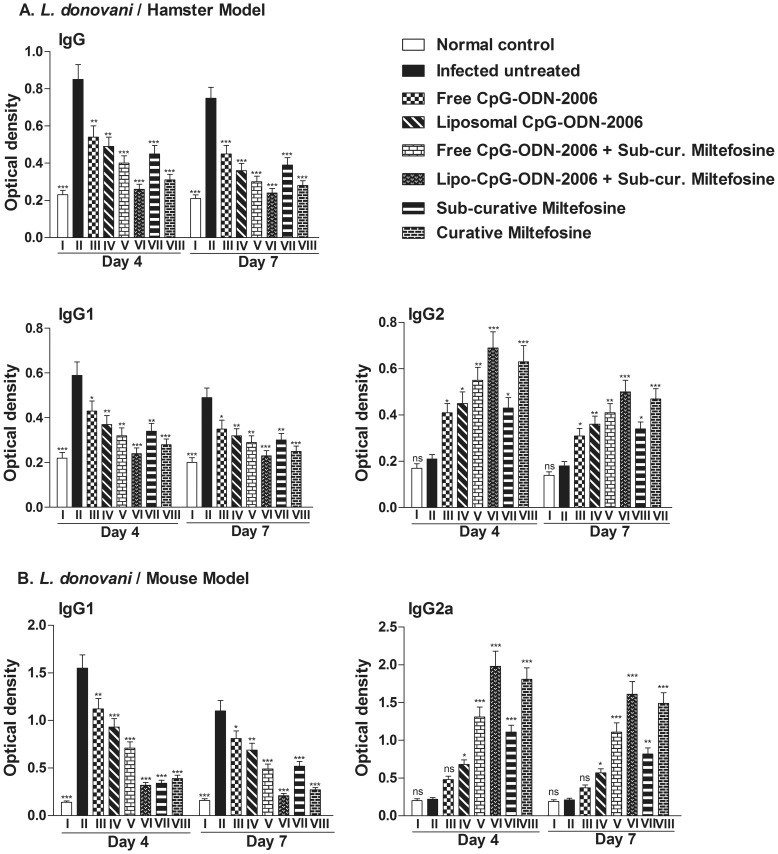
Effect of combination therapy on production of *Leishmania*-specific IgG and its isotypes, IgG1 and IgG2. Animals (n = 5/group) were treated with various combinations of CpG-ODN-2006 and miltefosine as described in legend of Figure 1. Sera collected from normal, infected and treated animals were processed by ELISA as described in materials and methods section to determine the (A) IgG, IgG1 and IgG2 production in hamsters and (B) IgG1 and IgG2a production in mice. Mean ± SD were calculated by the comparison of treated groups to its infected counterparts. The data are representative of three independent experiments. Each experiment was set in triplicates. The significance between various treated groups was calculated by one way ANOVA followed by Tukey's post test using graph pad Prism (version 5.0). Significance: Group II vs normal and all treated groups (*P<0.05, **P<0.01, ***P<0.001 and ns; non-significant). The data presented here are representative of two independent experiments.

Apart from subdued T-cell immune response, VL progression is also associated with involvement of *Leishmania*- specific antibody responses [34]. It has been reported earlier that *in vivo* administration of CpG-ODNs skew antigen-specific IgG isotype to IgG2a and promote the induction of antigen specific CD8^+^ cytotoxic T-cells in murine model of cutaneous leishmaniasis [35]. In our experiments, *Leishmania* specific IgG and its isotype IgG1 were elevated in sera of *Leishmania* infected hamsters (Group II) at both time points (day 4 and day 7 post treatment) along with decrease in levels of IgG2 isotype (Figure 4A). On the contrary, when compared with untreated control, the levels of IgG and IgG1 were found to be significantly decreased while the level of IgG2 was significantly increased in all the treated groups (Figure 4A). Among all the treated groups, combination of lipo-CpG-ODN-2006 and miltefosine (Group VI) showed maximum reduction (p<0.001) in the level of IgG (95.2% and 94.4% decrease) and isotype, IgG1 (94.6% and 98.1% decrease) and highest induction in the level of IgG2 (3.2 and 2.7 fold), when compared with infected untreated group at day 4 and 7 post treatment, respectively (Figure 4A). In mice also, IgG2a and IgG1 are used as surrogate markers for Th1 and Th2 responses as IgG2a levels are dependent on IFN-γ, whereas IgG1 levels correlate with IL-4 [36]. Similar to hamsters, significantly increased level of IgG1 and decreased level of IgG2a in the sera of infected untreated group (Group II) of Balb/c mice was observed which was completely reversed in all the treated groups where we have observed pronounced reduction in the level of IgG1 (>87% decrease in comparison to infected control) and rise in IgG2a antibody in lipo-CpG-ODN-2006 and miltefosine treated group (9.1 fold increase in comparison to infected control) (Figure 4B). Here too, combination of lipo-CpG-ODN-2006 and miltefosine (Group VI) showed maximum reduction (P<0.001) in the level of IgG1 and elevation (P<0.001) in the IgG2a level.

## Discussion

Host immune response has a direct impact on the efficacy of chemotherapy against leishmaniasis and one of the major complications for the chemotherapeutic treatment of VL is the depressed immune function exhibited by patients. Therefore, augmentation of protective immune responses in the infected host with immunomodulatory agents is essential for overcoming immunosuppression. Combination therapy using immunomodulator with standard chemotherapeutic regimen is already in practice for the treatment of tuberculosis [37] leprosy [38] and malaria [39]. Numerous reports have suggested use of CpG-ODN as an immunomodulator to boost host immunity based on its recognition by a variety of immune cells in various pathophysiological conditions [18], [19]. In leishmaniasis too we have earlier demonstrated that co-administration of lipo-CpG-ODN-2006 with sub-curative miltefosine increases the efficacy at short dose and time [23]. In the present study, we have shown that lipo-CpG-ODN-2006 in combination with sub-curative dose of miltefosine enhanced host immunological responses that helped in reducing parasite load. Although mouse model for *L. donovani* has been widely studied but it suffers from the limitation that it does not reproduce all the features of active human VL. In mouse model of VL, there is an early increase in parasite burden and after 6–8 weeks the infected animals elicits antileishmanial immune responses that controls the parasite multiplication. Even though Balb/c mice differ from symptomatic human subjects with *L. donovani* infection, they are good experimental model for early parasite replication and immune responses study [40], [41]. In contrast, the clinicopathologic features and immunopathologic mechanisms of VL in the hamster model are remarkably similar and closely mimic active human disease [41]. Systemic infection of the hamster with *L. donovani* results in relentless increase in visceral parasite burden, progressive cachexia, bone marrow dysfunction and ultimately death [42], [43], [44]. Considering these factors we have used both rodent models for our experimental infection and immune responses study following combination therapy. In non-healing *Leishmania* infection males develop stronger cell-mediated (Th1) responses while females develop stronger humoral (Th2) responses suggesting that males are more resistant to infection. However, conflicting reports also exist as in case of visceral leishmaniasis whether caused by *Leishmania donovani* in humans [45], [46] or *Leishmania infantum* in dogs [47] susceptibility is higher in males than females. Similarly, in case of *L. donovani* infection in hamster model, male developed more parasites than females [48] which decreased by castration while ovarectomy in females promoted infection [49]. Similar differences also exist in different mouse strains, while male Balb/c and DBA/2 mice are more susceptible to systemic infection with *Leishmania major* following intravenous inoculation [50] as compared to females; male C57Bl/10 and DBA/2 mice are more resistant than females to subcutaneous challenge with this parasite [51], [52]. Undoubtedly, the parasite species initiating infection, the tissue site involved and the host species are amongst the variable factors influencing these observed differences. In light of these conflicting reports, we included both sexes of mice and hamsters in our study and only those animals were included that had grade I infection (i.e. 5–10 amastigote/100 nuclei of liver or spleen cells). Our results demonstrated that inhibition of parasite burden during combination therapy with lipo-CpG-ODN-2006 plus sub-curative dose of miltefosine in both rodent models is associated with induction of strong cell-mediated immune responses including Th1 cytokine synthesis, NO generation, and robust lymphocyte proliferation along with induction of *Leishmania*- specific antibody responses.

Several reasons limit the use of miltefosine monotherapy. Some of these include teratogenic effect in pregnant women, fear of resistance and its long half-life (about 150 h) in humans [53], [54]. In contrast, combination therapy is advantageous over monotherapy as it delays or prevents the emergence of resistance and requires lower and shorter dose regimen against various infectious diseases. In the present study, we explored the synergy between chemotherapy and host immune function by using CpG-ODN-2006 in combination with miltefosine. Interestingly, assessment of splenic infection at later time point (at day 30 post treatment) in infected hamsters, that underwent combination therapy (Group VI), revealed almost complete absence of parasite burden in spleen cells (Figure S2), which further suggested that this therapy is also effective in chronic model of experimental VL. On a contrary, a moderate increase in parasite burden in hamsters treated with free and liposomal forms of CpG-ODN-2006 (12% and 10% increase, respectively at day 30 post treatment in comparison to day 7 post treatment) was observed. The increase in parasite burden was also examined in hamsters treated with sub-curative and curative doses of miltefosine, which showed 14% and 2% increase, respectively, on day 30 post treatment (Figure S2). Similar trend were also observed at day 30 post treatment in *L. donovani* infected Balb/c mice that underwent combination therapy (Figure S2). This piece of evidence also suggests that lipo-CpG-ODN-2006 with sub-curative miltefosine boosts host immunity which provides long term protection in chronic model of experimental VL. Moreover, synthetic bacterial ODNs with unmethylated CpG motifs are known to induce macrophage expression of IL-12, TNF-α and also activate NK cells to produce IFN-γ [55]. These cytokines (IL-12, IFN-γ and TNF-α) are involved in the development of Th1 dominated immune responses, which in context of VL is associated with disease suppression. Our data showed that lipo-CpG-ODN-2006 in combination with short dose of miltefosine has immense potential to skew Th2 type immune responses generated by *Leishmania* infection in hamsters towards host-protective Th1 response. IFN-γ, a hallmark Th1 cytokine was found to be significantly elevated in lipo-CpG-ODN-2006 plus sub-curative miltefosine treated animals along with IL-12. The synergistic stimulation of IL-12 with IFN-γ might provide a better additive effect for clearance of *Leishmania* parasite. The level of TNF-α mRNA expression was also increased in the group of animals treated with lipo-CpG-ODN-2006 plus miltefosine. VL progression is associated with induction of Th2- dominated immune responses in which, IL-10 has been demonstrated to antagonize Th1 driven cytokines, IL-12 and IFN-γ thereby blocking iNOS expression [15]. Along with IL-10, TGF-β also suppresses macrophage activation and generation of NO [15]. In line with this, our observation revealed that mRNA expression level of both Th2 cytokines, IL-10 and TGF-β, were down-regulated at both the time points in treated animals. The highest suppression of these cytokines was found to lipo-CpG-ODN-2006 plus sub-curative miltefosine treated group. Moreover, overall skewing of CD4^+^ T-cell differentiation in Th1 mode in infected animals, which undergo combination therapy with lipo-CpG-ODN-2006 plus miltefosine, has been further reflected in heightened expression of iNOS and generation of NO. This also supports the view regarding the up-regulation of iNOS by Th1 cell associated cytokines and confirms that NO-mediated macrophage effector mechanism is critical for controlling the parasites in the animal model [15].

Disease severity in experimental visceral leishmaniasis was associated with significantly hampered antigen presentation and antigen-specific T cell activation in murine and hamster models and elicitation of effective T-cell based host immune response defines the success of antileishmanial chemotherapeutics [28], [31], [32], [56]. In our study, enhanced antigen specific expansion of T-cell repertoire in all treated group of animals were observed with maximum induction in lipo-ODN-2006 and miltefosine (Group VI). This might be account for skewing the T-cell repertoire towards a Th1 type phenotype as substantiated by the elevated level of IFN-γ, IL-12 and TNF-α mRNA transcript in splenocytes derived from all treated group of hamsters and mice. Apart from liver and spleen, the involvement of lymph nodes has been well documented in clinical cases of leishmaniasis [57]–[60] as well as in experimental animals [61], [62] in the form of lymphadenopathy with occasional demonstration of leishmanial parasites. However, few studies in *Leishmania* infected experimental models have been conducted on lymph node involvement and the fate of lymph nodes during disease progression. Since lymph nodes are enriched in T-cell population that are migratory in nature [63], we also studied the proliferation status of lymphocytes in the lymph nodes of all groups of hamsters (i.e. chronically infected hosts) against the mitogen ConA and SLA. Here also a parallel outcome was observed similar to T-cell proliferation in splenocytes, as animals treated with combination of lipo-CpG-ODN-2006 and miltefosine (Group VI) showed maximum lymphoproliferative response in lymph nodes on day 5 post treatment of SLA thereby suggesting general induction of host protective response in secondary lymphoid organ following our combination therapy (see supplementary Figure S1). Apart from impaired cell mediated immunity, active VL is also associated with high levels of the *Leishmania* specific antibodies (Abs) observed before the detection of parasite specific T- cell response [64]. Unlike mice, where IL-4 and IL-12 dictates IgG subclass switching to IgG1 and IgG2a, respectively, such distinct IgG classes remain obscure in hamsters [65], [66]. It has been well documented that levels of IgG and IgG1 Abs increases with the parasite load in VL, whereas, the increase in IgG2 Ab is indicative of the development of effective immune responses [67]. The progressive increase in antileishmanial IgG2 and decrease in IgG/IgG1 in all the treated groups, with lipo-CPG-ODN-2006 plus miltefosine showing the highest difference, suggest that protection against leishmaniasis is induced by a strong T- cell response, which has been reported in both clinical and experimental VL [30]. Interestingly, in our *in vitro* study on extracellular promastigotes, we found that CpG-ODN and its liposomal preparation had no direct effect on the viability of extracellular form of parasite as judged by luciferase assay in transgenic promastigotes expressing luciferase as a part of episomal vector [68] (data not shown). This further strengthened the hypothesis that CpG-ODN-2006 only act through activation of host immunity which suppresses the parasite multiplication *in vivo* in both hamster and murine model of VL.

Development of strong cell-mediated immunological responses, like Th1 biased cytokine synthesis, iNOS induction and NO generation along with induction of *Leishmania* specific antibody responses (IgG2 elevation) resulted in disease resolution of VL. In our study, combination of lipo-CpG-ODN-2006 and sub-curative miltefosine showed almost complete suppression of parasite burden in infected animal on day 7 post treatment which was lesser at day 4 post treatment. This hold true for infected animals treated with curative dose of miltefosine and similar trend was observed for other treated group also. Reduction in parasite burden at day 7 post treatments in all treated group might be due to persistent effect of heightened cell mediated immune response observed even at day 4 post treatment which helped in amelioration of parasites from infected organs. Interestingly, in our study, host protective immune response was found to be diminished in all treated groups just after 3 days i.e. day 7 post treatment although it was still significantly higher than infected group (p<0.001). This is not surprising as reduced parasite burden at day 7 post treatment in all treated group might account for diminished levels of host protective immunological response which might be sufficient enough for elimination of rest of the parasite from both spleen and liver. The low levels of Th 1 cytokines, iNOS, NO and IgG2 at day 7 post treatment is thus consistent with the decreasing parasite loads seen in the treated group. Overall, our findings demonstrate that liposomal CpG-ODN-2006 enhances host immunological response thereby minimizing both the dose and duration of miltefosine during combination therapy in animal model of VL. Establishment of appropriate cell mediated immunity using this immunomodulator might be helpful for not only treatment of non healing form of leishmaniasis, but also for treatment of other chronic macrophage associated infectious diseases.

## Supporting Information

Figure S1 Effect of CpG-ODN-2006 and miltefosine combination therapy on lymphocyte proliferation at day 4 and 7 post treatments. Lymph nodes were isolated from all the groups of hamsters (normal, infected and treated) and incubated in the presence and absence of concanavalin A (5 µg/mL) or soluble *Leishmania* antigen (10 µg/mL) at 37°C for 72 h and lymphocyte proliferation was assessed by using XTT dye. Proliferation is represented as ratio of mean optical density (OD) of stimulated culture/unstimulated control. Three independent experiments were done and each bar represents pooled data (mean ± SD) of five hamsters and the data represent the means of triplicate wells. Mean ± SD between different groups were calculated by one way ANOVA followed by Tukey's post test using graph pad Prism (version 5.0). Significance: Group II vs normal and all treated groups (*P<0.05, **P<0.01, ***P<0.001 and ns; non-significant).
(TIF)

Figure S2 Effect of CpG-ODN-2006 and miltefosine combination therapy on long term protection against VL. *L. donovani* infected mice and hamsters were treated with various combinations of CpG-ODN-2006 and miltefosine as described in materials and methods section. At day 30 post treatment animals of different experimental groups were sacrificed and the splenic parasite load was determined by stamps-smear method. Total parasite load in each organ is expressed in LDU unit. Data represents here are representative of three independent experiments. Each of the experiments was done a minimum of three times and data represents mean ± SD. The significance between different experimental groups was calculated by one way ANOVA followed by Tukey's post test using graph pad Prism (version 5.0). Significance: Group II vs normal and all treated groups, group V vs VI and group V vs VII (**P<0.01, ***P<0.001).
(TIF)
