# Supplementary figures and images for: Combination of Liposomal CpG Oligodeoxynucleotide 2006 and Miltefosine Induces Strong Cell-Mediated Immunity during Experimental Visceral Leishmaniasis
Source: PLoS One. 2014 Apr 14;9(4):e94596. doi: 10.1371/journal.pone.0094596 (PMC3986403; doi:10.1371/journal.pone.0094596)

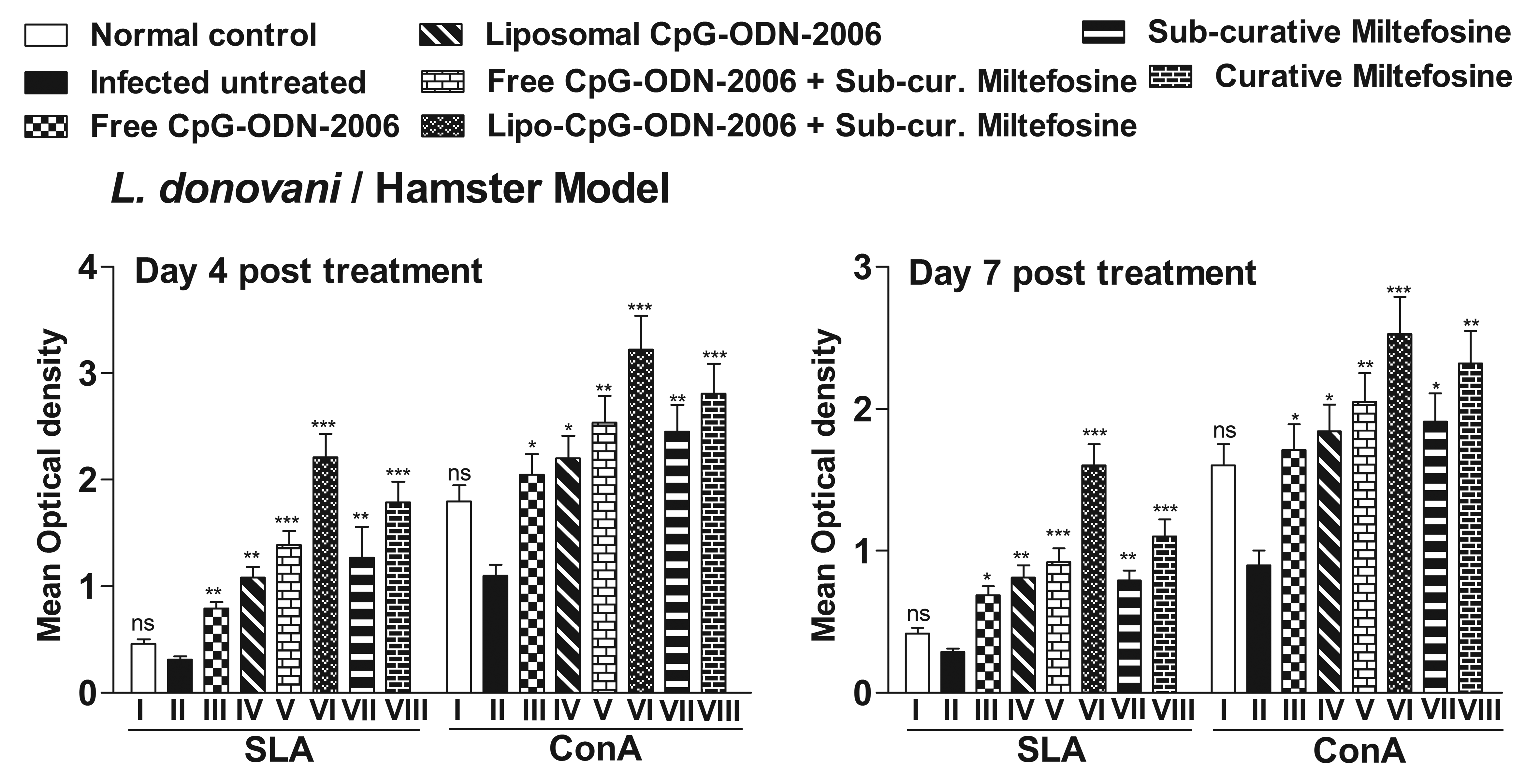

Supplement: Figure S1 — Effect of CpG-ODN-2006 and miltefosine combination therapy on lymphocyte proliferation at day 4 and 7 post treatments. Lymph nodes were isolated from all the groups of hamsters (normal, infected and treated) and incubated in the presence and absence of concanavalin A (5 µg/mL) or soluble Leishmania antigen (10 µg/mL) at 37°C for 72 h and lymphocyte proliferation was assessed by using XTT dye. Proliferation is represented as ratio of mean optical density (OD) of stimulated culture/unstimulated control. Three independent experiments were done and each bar represents pooled data (mean ± SD) of five hamsters and the data represent the means of triplicate wells. Mean ± SD between different groups were calculated by one way ANOVA followed by Tukey's post test using graph pad Prism (version 5.0). Significance: Group II vs normal and all treated groups (*P<0.05, **P<0.01, ***P<0.001 and ns; non-significant). (TIF) [file pone.0094596.s001.tif]

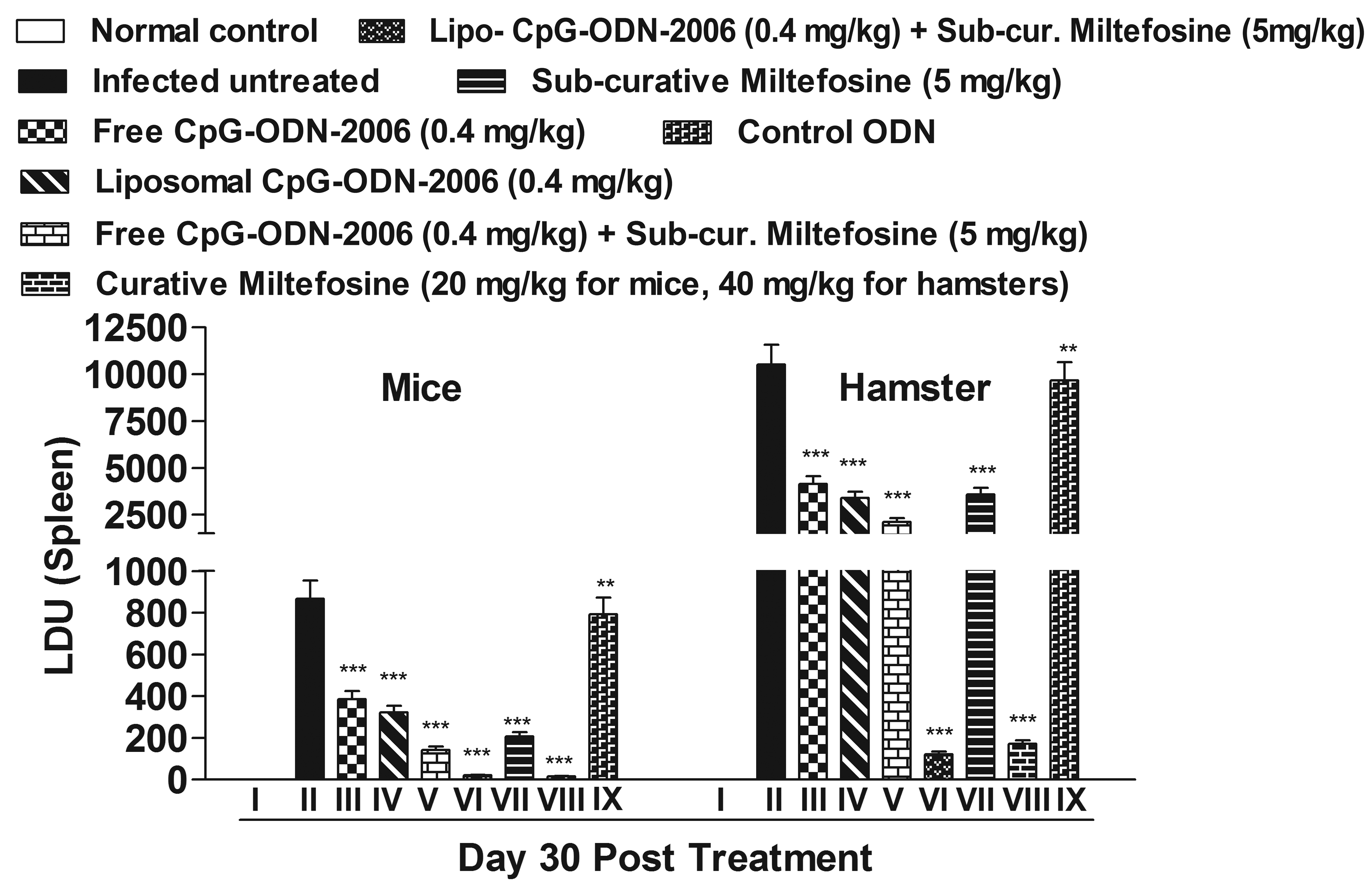

Supplement: Figure S2 — Effect of CpG-ODN-2006 and miltefosine combination therapy on long term protection against VL. L. donovani infected mice and hamsters were treated with various combinations of CpG-ODN-2006 and miltefosine as described in materials and methods section. At day 30 post treatment animals of different experimental groups were sacrificed and the splenic parasite load was determined by stamps-smear method. Total parasite load in each organ is expressed in LDU unit. Data represents here are representative of three independent experiments. Each of the experiments was done a minimum of three times and data represents mean ± SD. The significance between different experimental groups was calculated by one way ANOVA followed by Tukey's post test using graph pad Prism (version 5.0). Significance: Group II vs normal and all treated groups, group V vs VI and group V vs VII (**P<0.01, ***P<0.001). (TIF) [file pone.0094596.s002.tif]
